# In house virtual surgery and 3D complex head and neck reconstruction

**DOI:** 10.1186/s40463-018-0320-9

**Published:** 2018-12-12

**Authors:** Kimberly Luu, Amirreza Pakdel, Edward Wang, Eitan Prisman

**Affiliations:** Division of Otolaryngology, Head & Neck Surgery, University of British Columbia, 2775 Laurel Street, DHCC 4th. Floor, Vancouver, BC V5Z 1M9 Canada

**Keywords:** Stereolithography, Mandibular reconstruction, 3D printing, Surgical cutting guide

## Abstract

**Background:**

3-Dimensional (3D) printing can be applied to virtual planning and creation of surgical guides for mandibular reconstruction. Such systems are becoming increasingly prevalent in head and neck reconstruction. However, third party access to this technology is costly and removes the opportunity to design, create, and modify the bony reconstructions, as third party technology is a black box. This series is a pilot study to document the feasibility of an in-house software tool. The objectives of this study are to describe the design of an automated in house system and assess the accuracy of this in house automated software tool for mandibular reconstruction in a simulated environment.

**Methods:**

Software was written to automate the preoperative planning and surgical guide creation process. In a simulation lab, Otolaryngology residents were tasked with resecting and reconstructing a simulated mandible using the 3D-printed cutting guides. A control group of residents performed resection and reconstruction using the traditional method without cutting guides. T-test analysis was performed to compare specific aspects of the final reconstructions including: change from native mandibular width and projection, segment gap distance, and reconstruction time.

**Results:**

Mandibular reconstruction was successful in all participants using the 3D printed system.

The guided group performed significantly better on the measurement of change in Mandibular overlap, projection, segment gap volume. There was a non-significant trend towards better mandibular width and operative time for the guided group.

**Conclusions:**

This study confirms functionality and feasibility of using an in house automated software for planning and creating surgical guides.

## Background

Advanced head and neck malignancies with underlying bony involvement often require aggressive oncological resection of large segments of the oral cavity including the mandible or maxilla. Reconstruction following these ablations is provided by microvascular transplantation of osseo-cutaneous tissue such as a fibular or scapula free flap [1]. These transplanted free flaps require multiple osteotomies in complex 3-dimensional (3D) orientations in order to reconstitute the premorbid bony and soft tissue anatomical structures of the head and neck. The accuracy of the reconstruction has significant impact on cosmetic and functional outcomes such as mastication, articulation, deglutition and breathing, as well as considerable effect on the patient’s quality of life.

Traditional intraoperative execution of these complex reconstructions continues to be challenging and time consuming. This technique may be improved with advanced preoperative planning. One such approach, which has been used in a variety of craniofacial reconstructions utilizes 3D printed objects that are generated with patient-specific geometrical data from computed tomography (CT) scans [2]. Potential benefits of preoperative planning include reducing operative time and cost, improving patient understanding, resident education, and refining surgical predictability and outcomes [3]. Ultimately, this innovation has the potential to lead to a decrease in patient morbidity and an improved quality of life after having undergone head and neck reconstruction.

Studies on the use of 3D printing in head and neck reconstruction currently uses third party commercial software. Commercial software is expensive and the lack of direct access to the technology and immediate feedback creates a tedious preoperative planning process. We postulate that technology designed and created in house will provide the benefits of commercial software in terms of preoperative virtual planning and accurate reconstructions, while reducing the cost. Additionally, continuous improvements can be made on the technology, guided by direct user experience. Future innovations, such as the ability to design one step dental implants, can be planned and implemented. The purpose of this study is to describe and pilot an in-house software to produce three-dimensional surgical guides for use in mandibular reconstruction. The objectives of this study are to describe the design of an automated in house system and assess the accuracy of an in house automated software tool for mandibular reconstruction in a simulated environment.

## Methods

### Mandibular guide development

In house software for preoperative virtual planning and creation of surgical guides was designed and developed through collaboration between the principal investigator, a head and neck reconstructive surgeon and a biomechanical engineer. The preoperative surgical planning tool was developed with Amira v5 software (FEI Visualization Sciences Group, Berlin, Germany) for visualization of CT data, which was bridged with the automation algorithm written in MATLAB (Mathworks, Natick, MA)[4]. The process is based on the predetermined principles and begins with the input of a DICOM CT scan image. A 3D reconstruction of the mandible is generated by automatic segmentation based on Hounsfield unit thresholds of cortical bone (~ 400 HU), with subsequent refinement input from the user to remove any aberrations (e.g. due to tooth filling metal artefacts, segmentation defects due cartilaginous TMJ tissue etc.).

Resection margins are then indicated with 3D cutting planes on the virtual model through an interactive user interface. Next, the desired location of the mandibular plate is drawn free-hand on the outer cortical surface of the mandible, extending from the subcondyle surface, though the angle and body, and ending at the contralateral para-symphyseal surface. The reconstruction is then automatically planned based on a custom implementation of the Ramer–Douglas–Peucker contour simplification algorithm. This process optimizes the mandibular contour and bony apposition of the osteotomies by reconstructing the 3D curved geometry of the resected mandibular segment as a series of straight segments. A number of constraints can be defined, such as the desired number of osteotomies, the minimum and maximum length of each segment. A critical component of the algorithm is automatic determination of the cutting angle for each segment of the donor bone to achieve maximal opposition when the segments are linearly arranged.

**Fig. 1:**
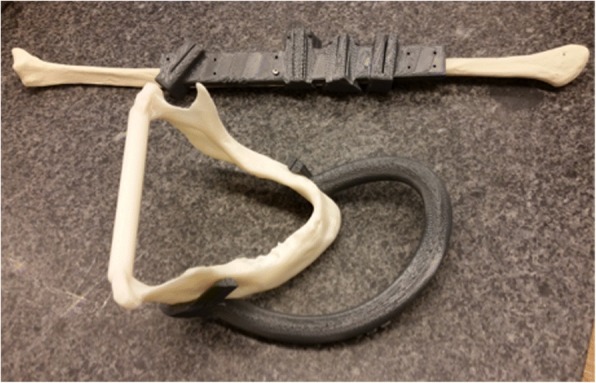
The 3D printed surgical guides attached to the diseased mandible and generic fibula. Picture of the 3D printed surgical guides and 3D printed disease mandible. It shows how the surgical guides would attach onto a mandible. The surgical cutting guide is also attached to a generic plastic fibula

Once the resection and reconstruction is planned, the software displays the reconstructed mandible with the fibular segments for a final check. Virtual models of the surgical cutting guides are then created with the reverse image of the mandibular model so the guide can snap on at the appropriate place during the operation. Each guide houses a slit that corresponds to the cutting plane that will reproduce the virtually planned resection on the mandible, and the planned sectioning on the fibula. Figure 1 shows the 3D printed mandible and surgical cutting guides.

### Experimental method

The institutional research ethics board approved the study protocol. Otolaryngology residents from the University of British Columbia were invited to participate in the study during a bone-plating simulation day. The residents all received a demonstration and practice time for performing mandibular reconstruction on the simulated models.

Residents were randomized into a control group, which performed the reconstructions by a traditional free-hand method and a guided group, which performed the reconstructions with surgical cutting guides. The randomization was blocked by residency training year. Each resident had their own workstation and were not able to see the reconstructions of the other participants. Both groups were tasked with resecting and reconstructing a real case of an oral squamous cell carcinoma invading the mandible.

All participants were provided with a 3D print out of the diseased mandible and a generic plastic fibula. The free-hand group designed and performed the mandibular resection and reconstruction using a ruler as a guide [5]. The participants marked out the osteotomy sites on the fibula, including the angle at which to make the cuts. The group was allowed to make as many adjustments as they felt were needed. The guided group was provided plastic guides designed to lock onto the mandible and generic fibula with slots that guided the resection and osteotomies. Once they were all made, the participants plated the fibular sections. The reconstructed mandibles were then anonymized.

### Analysis

All reconstructions were classified as successful if mandibular continuity was achieved. All participants recorded the amount of time required to perform the reconstructions.

**Fig. 2:**
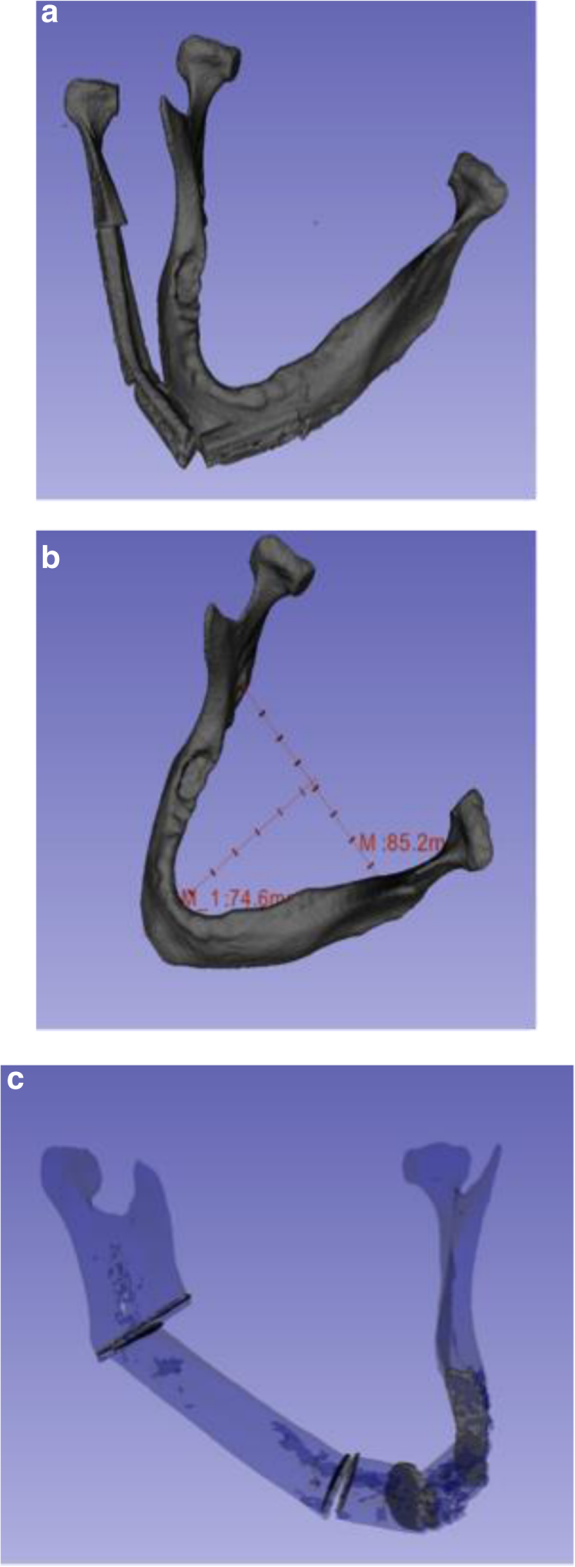
Measurements of accuracy including overlap, projection, width, and gap volume between segments. The reconstructed mandibles were CT scanned and segmented to create models. Mathematical calculations were then performed on the models to determine accuracy. These pictures show which measurements were taken to calculate **a**. overlap between native and reconstructed mandible, **b**. the change in projection and width, an **c**. the gap volume between each segment of the reconstructed mandible

CT scans were taken of each reconstruction and a 3D print out of the original mandible. Using the open source medical imaging software 3DSlicer version 4.5 [6], triangular mesh models were rendered from the CT scans. Accuracy was assessed by a number of measurements shown in Fig. 2 including: overlap of the reconstruction with the native mandible, volume of each gap between osteotomies, the change in width and projection of the mandible.

### Volume overlap

The volume overlap was calculated using the Model-to-Model Distance extension in 3DSlicer [7]. The mean distance between the two models on a point-to-point basis was used as the value of comparison.

### Gap analysis

The volume of the gap was estimated by subtracting the convex hull (volume) of each individual segment from the convex hull of the two opposing segments. This algorithm was provided in 3DSlicer.

### Width and projection

The width was defined as the distance between each condyle (point A and B) and the projection was defined as the distance from the midpoint of AB and the most anterior part of the reconstruction.

Data was analyzed using SPSS. The mean results of the above measurements between the guided and nonguided groups was compared with a 2-sample paired t test. Comparison of the time to completion for each mandicular reconstruction between the guided and unguided group was done with another 2-sample t test. Statistical significance was set at *p* < 0.05.

## Results

**Fig. 3:**
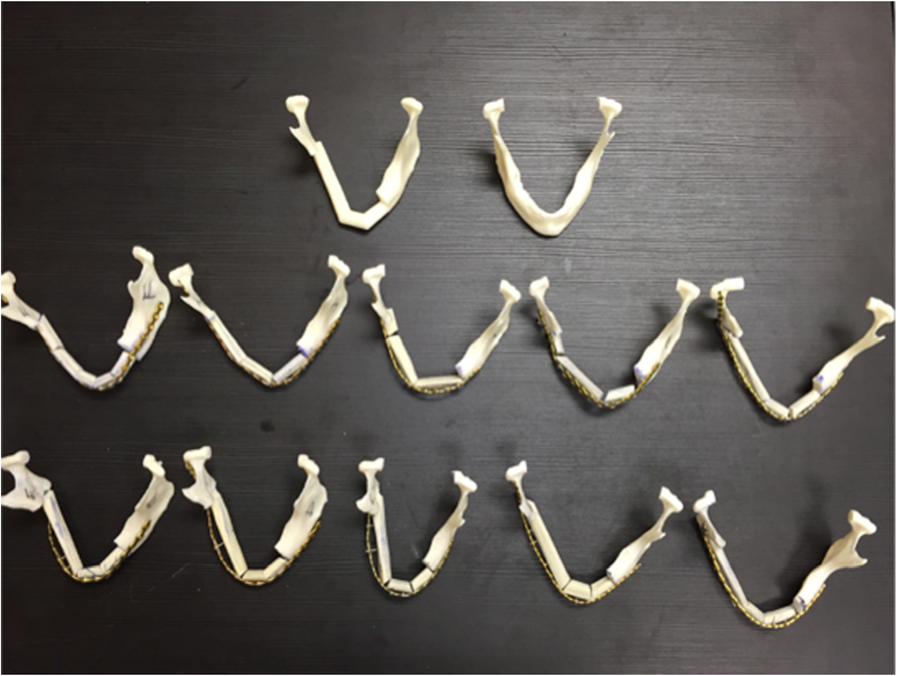
The anonymized reconstruction results with the native mandible and printed optimal virtually planned reconstruction. This picture show all the reconstructed mandibles with a 3D print out of the original diseased mandible and a printout of the virtually planned reconstruction

A total of 10 residents participated in the study. There were two residents in each training year from PGY1 – PGY5. The residents in each year had a comparable amount of experience in mandibular reconstruction and all participated in the morning plating course. All residents completed reconstruction in the group they were allocated. All mandibular reconstructions achieved continuity. Figure 3 displays the final reconstruction results for each participant.

### Time to completion

**Fig. 4:**
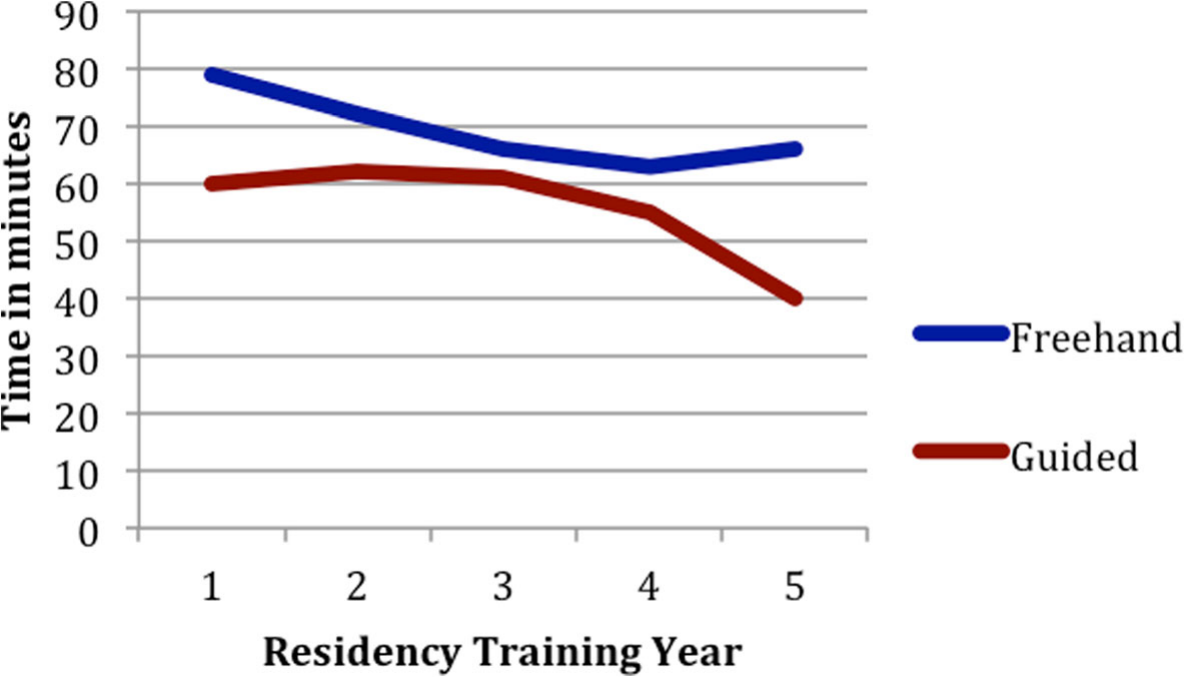
A non-significant decrease in time to completion was shown between the freehand and guided group. This graph shows the time to completion of the reconstructions with 1 line for the freehand group and 1 line for the guided group. There is a non-significant trend to decrease in time for the guided group. Additionally, residents in higher training years performed the reconstruction in less time as expected

Average time for completion of reconstruction was less for the guided group but not statistically significant. Figure 4 shows the time for completion of each resident There is an inverse relationship between time to completion and residency training year, as expected. Average time to completion without bone plating time for freehand and guided group was 51 min and 46 min respectively with *p* = 0.172.

### Computed tomography analysis

**Fig. 5:**
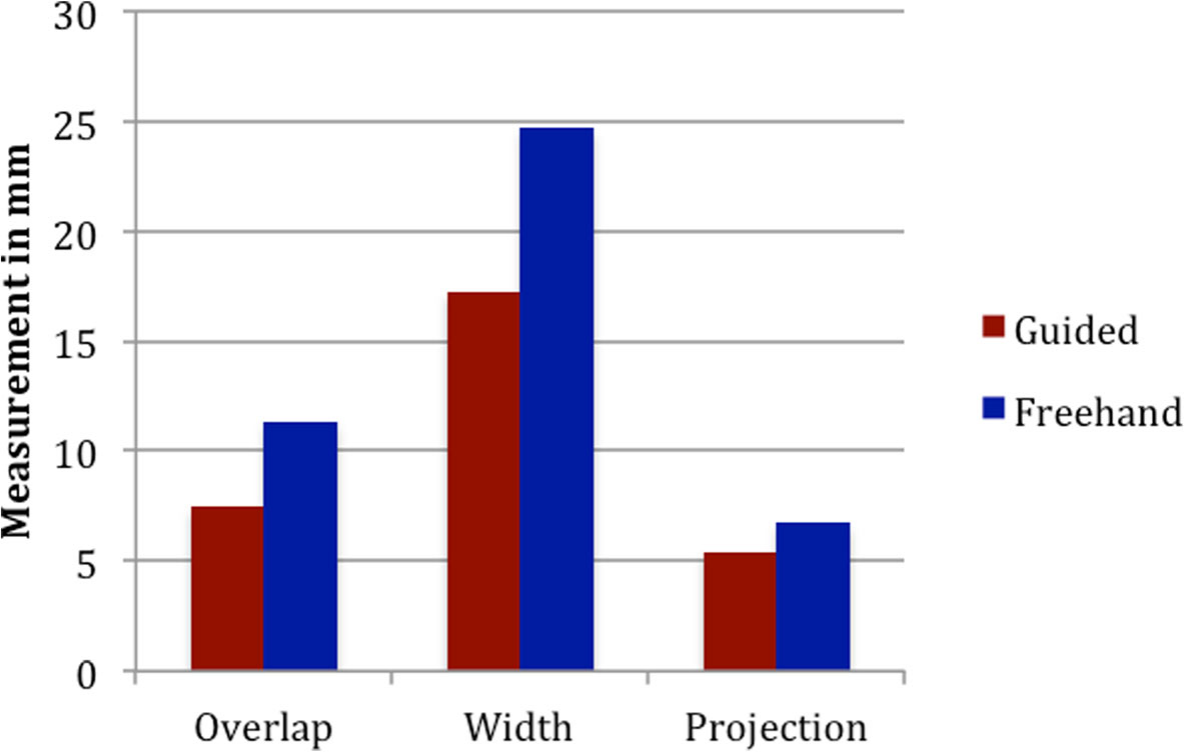
A significant decrease in the differences between the native and reconstructed mandible was shown for overlap and width. This graph compares the accuracy measurements for the reconstructions performed freehand with the reconstructions performed with the guide. There shows a decrease in the change of projection and width with the guided group

**Fig. 6:**
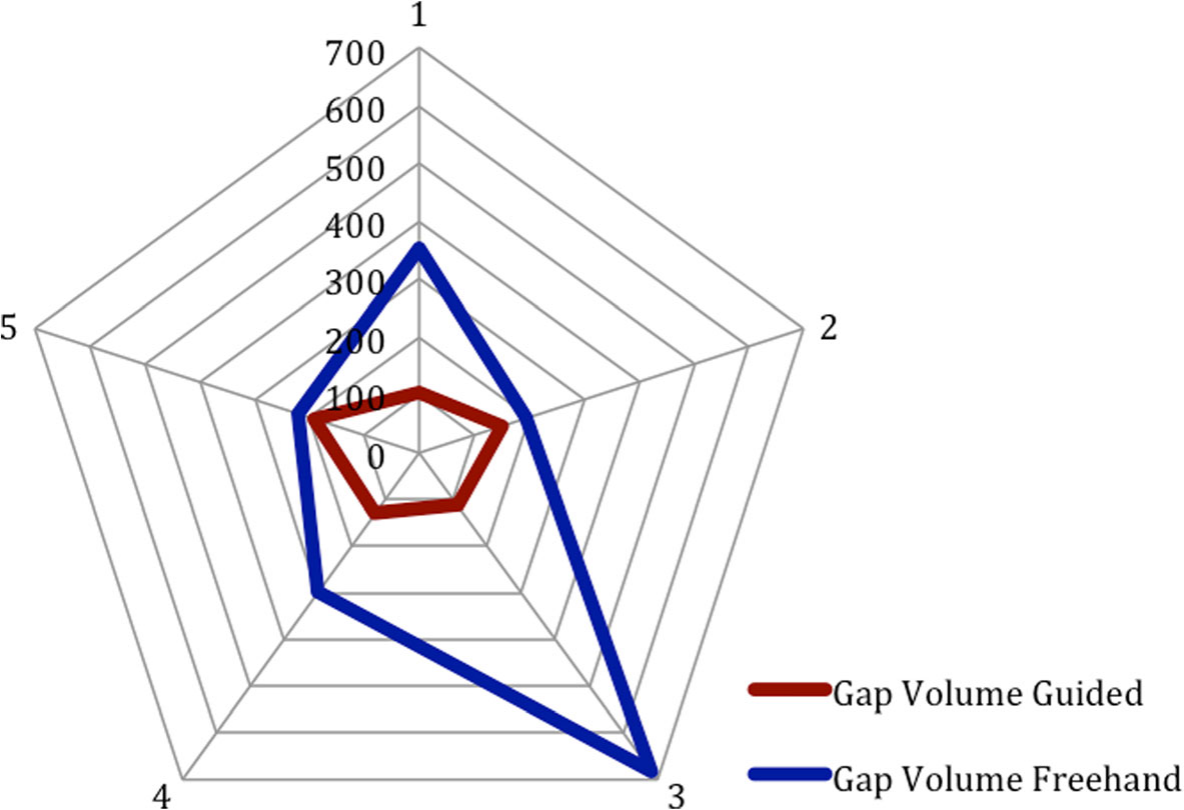
The volume of the gap between osteotomies was significantly decreased in the guided group compared to the freehand group. This figure shows the average volume of the gaps between segments in the guided and freehand group. The volume of gap is greater in the freehand group. This is an important outcome, as a surgeon would want to optimize the bony contact to increase healing

Results show that the guided group performed better in all categories. Table 1 details the results of the measurements for each participant. Figures 5 and 6 graphically depicts the significant improvement in gap volume, overlap, and projection.

**Table 1:** Results of accuracy measurements of the guided and nonguided groups

Overlap Guided	Overlap Nonguided	Gap Guided	Gap Nonguided	Width Guided	Width Nonguided	Projection Guided	Projection Nonguided
(mm)	(mm)	(mm3)	(mm3)	(mm)	(mm)	(mm)	(mm)
7.89	9.96	104.53	354.73	20.1	20.9	2.3	5.3
10.47	12.22	150.37	194.33	24.2	33.5	4.3	6.8
8.65	12.15	112.52	683.24	21.6	23.7	9.8	7.1
2.49	6.89	129.31	297.27	7.3	13.1	7.1	4.1
7.75	15.45	191.58	220.04	13.2	32.2	3.6	10.3

The detailed accuracy measurements of the overlap, gap, width and projection of the guided and nonguided reconstructions

## Discussion

This study aimed to demonstrate feasibility and accuracy of surgical cutting guides created with an in-house software for mandibular reconstruction. The in house software was able to create surgical guides with minimal intervention from the operating surgeon. Time to completion of the guided versus freehand group was faster, but not statistically significant. The guided group performed significantly better in creating reconstructions with appropriate mandibular projection and lesser segment gap distant. There was a non-significant trend towards better mandibular width.

The majority of 3D printing for mandibular reconstruction is done with third party software [8, 9]. The cost of commercial software from KLS Martin Group or Synthes can range from $4000 - $15,000 per case compared to $100 - $200 per case using this in house software. In addition to cost, the workflow of commercial software is often not ideal. It may take several interactions and face-to-face meetings the company to finalize a preoperative plan, making additional changes difficult and time consuming. Dupre-Bories et al. have also made an effort to create the own in house technology to overcome this inefficient. The group has shown a reduction of cutting guide delivery time from 21 to 5 days [10]. In contrast, the technology described in this paper delivers a cutting guide in 2–3 days. The time gain stems from improved workflow as well as increased automation in the guide creation process. This automation removes multiple steps in which the user has to interact with the software or a designer. In the future, this automation can allow for intraoperative changes to be made if unexpected changes, such as tumour progression, are encountered.

Multiple studies have shown the use of 3D printed surgical cutting guides for mandibular reconstruction is feasible [11–13]. The most common measurable benefit that has been shown is intraoperative time gain [14–16]. In the simulated environment, our study did not show a significant decrease in operative time. This could be explained by a decrease in complexity from a simulated environment that lacks soft tissue and challenges with exposure, bleeding, or access and the small number of participants in the study. Many additional benefits of virtual planning have been hypothesized, but have not been consistently proven. This pilot study demonstrates the use of appropriate objective measurements that can assess the accuracy of mandibular reconstruction and demonstrated an improvement in accuracy. A clinical trial can use the same accuracy assessment protocol.

The design and development of the in house software resulted in a number of learning points. There was discussion around what factors should be optimized in the reconstruction when developing the surgical guides. Lower contour of the mandible provided the best cosmetic outcome, but optimizing for upper alveolus allowed the design to remain flexible for the future implementation of one-stage dental implants. The optimization of contour would most theoretically lead to an infinite number of osteotomies. The authors had to decide what practical constraints to include in the algorithm in order to consider bone survival, operative difficulty, and operative time. The use of an in-house software affords the author the ability to consider these variables and systematically test their influence on reconstruction outcome.

This paper has a number of limitations. The participants are residents who are not the primary surgeons in many of these complex reconstructive cases. Additionally, they are of varying years and a low number, both of which would influence the significance of the results. Novice surgeons would likely benefit more from the use of cutting guides so the increase in accuracy may be exaggerated. The residents were given time to practice plating skills to introduce the simulated environment and provide all participants with baseline skills. However, more practice would have put the residents at an equal skill level and improved the validity of the comparison. Given these limitations however, the guided group were all still able to complete the reconstruction, showing feasibility of the technology that is generalizable to real practice.

## Conclusion

This pilot study confirmed the feasibility of designing and developing an in-house software to automate mandibular reconstruction for head and neck cancer resections. Mandibles reconstructed with the surgical guides have comparable accuracy when compared to traditional reconstruction in terms of overlap, volume between each osteotomy gap, and the width and projection of the mandible.

## Abbreviations

3D: 3 dimensional
CT: Computed tomography

## References

[1] Mehta RP, Deschler DG (2004). Mandibular reconstruction in 2004: an analysis of different techniques. Curr Opin Otolaryngol Head Neck Surg.

[2] Anuja A, Chen W, Kolokythas A (2011). Use of virtual surgery and Stereolithography-guided osteotomy for mandibular reconstruction with the free fibula. Plastic & Reconstructive Surgery.

[3] Chow LK, Cheung LK (2007). The usefulness of stereomodels in maxillofacial surgical management. J Oral Maxillofac Surg.

[4] Matlab by Mathworks. https://www.mathworks.com (2017). Accessed Dec 2015.

[5] Kang S, Old M, Teknos T (2016). Contour and osteotomy of free fibula transplant using a ruler template. Laryngoscope.

[6] 3D Slicer. http://www.slicer.org (2017). Accessed 20 Feb 2016.

[7] Model to Model Distance. https://www.slicer.org/wiki/Documentation/Nightly/Extensions/ModelToModelDistance (2017). Accessed 23 July 2016.

[8] Cohen A, Laviv A, Berman P (2009). Mandibular reconstruction using stereolithogrpahic 3-dimensional printing modelling technology. Oral Surgery OralMedicine Oral Pathology Oral Radiology.

[9] Sannomiya EK, Silva JV, Brito AA, Saez DM, Angelieri F, Dalben GS (2008). Surgical planning for resection of an ameloblastoma and reconstruction of the mandible using a selective laser sintering 3D biomodel. Oral Surg Oral Med Oral Pathol Oral Radiol Endod.

[10] Dupre-Boris A, Vergez S, Meresse T, Brouillet F, Bertrand G. Contribution of 3D printing to mandibular reconstruction after cancer. Eur Ann Otorhinolaryngol Head Neck Dis. 2018;135(2):133-36. 10.1016/j.anorl.2017.09.007 29100719

[11] Cohen A, Laviv A, Berman P, Nashef R, Abu-Tair J (2009). Mandibular reconstruction using stereolithographic 3-dimensional printing modeling. Technology Oral Surg Oral Med Oral Pathol Oral Radiol Endod.

[12] Cunningham LL, Madsen MJ, Peterson G (2005). Stereolithographic modeling technology applied to tumor resection. J Oral Maxillofac Surg.

[13] Thomas CV, McMillan KG, Jeynes P, Martin T, Parmar S (2013). Use of a titanium cutting guide to assist raising the composite radial forearm free flap. Int J Oral Maxillofac Surg.

[14] Prisman E, Haerle SK, Irish JC, Daly M, Miles B, Chan H (2014). Value of preoperative mandibular plating in reconstruction of the mandible. Head Neck.

[15] Hanasono MM, Skoracki RJ (2013). Computer-assisted design and rapid prototype modeling in microvascular mandible reconstruction. Laryngoscope.

[16] Culie D, Dassonville O, Poissonnet G, Riss JC, Fernandez J, Bozec A (2016). Virtual planning and guided surgery in fibular free-flap mandibular reconstruction: a 29-case series. Eur Ann Otorhinolaryngol Head Neck Dis.

